# Diametric Role of the Latency-Associated Protein Acr1 of *Mycobacterium tuberculosis* in Modulating the Functionality of Pre- and Post-maturational Stages of Dendritic Cells

**DOI:** 10.3389/fimmu.2017.00624

**Published:** 2017-05-30

**Authors:** Mohammed Amir, Mohammad Aqdas, Sajid Nadeem, Kaneez F. Siddiqui, Nargis Khan, Javaid A. Sheikh, Javed N. Agrewala

**Affiliations:** ^1^Immunology Laboratory, CSIR-Institute of Microbial Technology, Chandigarh, India

**Keywords:** Acr1, *Mycobacterium tuberculosis*, immunomodulation, dendritic cells, Th1 cells, Th17 cells

## Abstract

It is instrumental for the *Mycobacterium tuberculosis (Mtb)* to persist within its host in dormancy. *Mtb* represses most of its metabolic machinery during latency, but upregulates the expression of latency-associated protein alpha-crystallin protein (Acr1). Therefore, it is imperative to understand how throughout dormancy, *Mtb* employs Acr1 to regulate the host immunity. This study reveals that Acr1 exhibits divergent effect on the pre- and post-maturation stages of dendritic cells (DCs). In the current study, we demonstrate that early encounter of bone marrow cells with Acr1 while differentiating into DCs (AcrDC^pre^), leads to impairment in their maturation. In contrast, when exposed to Acr1 after maturation (AcrDC^post^), DCs show augmentation in their activity, secretion of TNF-α, IL-12, IL-6, and activation of T cells. Additionally, AcrDC^post^ promoted the polarization of naïve CD4 T cells to Th1 cells and Th17 cells and restricted the intracellular growth of *Mtb*. Furthermore, these DCs upregulated the expression of CCR7 and exhibited enhanced migratory capabilities. The discrete impact of Acr1 on DCs is mediated through a mechanism involving STAT-1, SOCS-3, ERK, TLR-4, and NF-κB signaling pathways. This study reveals the unprecedented role of Acr1 in distinctly modulating the function of DCs at different stages of maturation.

## Introduction

One of the most striking features of *Mycobacterium tuberculosis* (*Mtb*) is its ability to survive in a state of dormancy within the hostile environment of host (1). Therefore, it is of the utmost importance to understand the factors that govern the bug’s survival and manage to subvert the host’s immunity. Some of the suggested strategies that *Mtb* exploits to tune host immunity are inhibition of phagolysosome fusion, impairment of innate immunity by obstructing pattern recognition receptors signaling, hampering the adaptive immunity by modulating the expression of costimulatory molecules, and production of cytokines along with exhaustion and apoptosis of T cells (2–7). Among the various proteins, alpha-crystallin protein (Acr1) is most dramatically upregulated during dormancy (8). *Mtb* devoid of Acr1 fails to maintain its latency (9), indicating that Acr1 is indispensable for the persistence of mycobacterium in dormant state.

Dendritic cells (DCs) play a cardinal role in imparting immunity against *Mtb* by activating and differentiating naïve T cells to effector and memory T cells. Upon infection with *Mtb*, DCs can initiate either inflammatory or regulatory responses that determine whether a pathogen will be cleared or retained (10). In response, *Mtb* can exploit its own machinery to inhibit T cell, B cell, and macrophage functions (6, 11).

Several proteins of *Mtb* like CFP-10 and ESAT-6 are reported to elicit the maturation of DCs and prime the T cell response (12, 13). In contrast, other investigators suggest that DCs differentiated in the presence of 10 kDa secretory antigen (MTSA) may provide a niche for the survival of mycobacterium (14). Recently, we have demonstrated that 16 kDa protein of *Mtb*, also known as Acr1/Rv2031c, inhibited the maturation of bone marrow cells (BMCs) to DCs (6). These DCs exhibited tolerogenic function and phenotype. Further, an interesting finding suggested an immunomodulatory role of Acr1 on differentiated DCs by suppressing tumor growth (15). This prompted us to investigate the impact of Acr1 on the differentiated DCs (AcrDC^post^) and their ability to stimulate T cells as well as to restrict the growth of *Mtb*. Furthermore, we compared AcrDC^post^ functionality with DC precursors that encountered Acr1 during their maturation (AcrDC^pre^). We observed that AcrDC^post^ exhibited stimulatory function as evidenced by the DC’s ability to activate and differentiate T cells and restrict the growth of *Mtb*. The mechanism suggested was through the modulation of STAT-1, ERK, and NF-κB pathways. In contrast, AcrDC^pre^ showed inhibitory function of DCs. This observation illustrates the diametric role of Acr1 in inhibiting the maturation of DCs precursor during early stage of differentiation, whereas the post-maturation encounter potentiates DCs development.

## Materials and Methods

### Animals

Female C3H/HeN, C3H/HeJ (*Tlr4^Lps-d^*), and C57BL/6 mice, 6–8 weeks, were procured from the Institute of Microbial Technology, Chandigarh, India. All the animal experiments were approved and complied by the Institutional Animal Ethics Committee (IAEC) and Regulatory Guidelines issued by the Committee for the Purpose of Supervision of Experiments on Animals (CPCSEA) (No. 55/1999), Ministry of Environment and Forest, Government of India.

### Antibodies and Reagents

Recombinant GM-CSF, IL-4, and naïve CD4 T cell enrichment kit, Abs to IL-12p40/70, TNF-α, IL-6, IFN-γ, IL-17, PE-Cy7-CD11c, FITC-CD80, PE-CD86, efluor-MHCII, APC-CD40, STAT-1, pSTAT-1, STAT-4, pSTAT-4, JNK, and pJNK were purchased from BD Biosciences (San Diego, CA, USA). Biotinylated anti-CD83 and APC-conjugated CCR7 Abs were purchased from eBioscience (San Diego, CA, USA). Abs against actin were from Sigma (St. Louis, MO, USA), SOCS-3 from Abcam (Cambridge, MA, USA) and anti-pERK and ERK Abs from Santa Cruz Biotechnology (Santa Cruz, CA, USA). The endotoxin removal column was purchased from Thermo Scientific (Rockford, IL, USA).

### DC Culture

Bone marrow cells were collected from femurs and tibia. The BMCs (2 × 10^6^/well) were cultured in media containing GM-CSF (2 ng/ml) + IL-4 (4 ng/ml) either with Acr1 (AcrDC^pre^) or without Acr1 (AcrDC^post^). On the third day, both cultures were replenished with media containing GM-CSF and IL-4, with Acr1 added only to the AcrDC^pre^ culture. On the sixth day, the AcrDC^post^ were stimulated with Acr1 (0–18 µg/ml) for 24 h, while the BMCs incubated with GM-CSF + IL-4 were used as controls (cDCs).

### Western Blotting

AcrDC^pre^ and AcrDC^post^ were harvested, washed, and lysed in lysis buffer (RIPA buffer, protease, and phosphatase inhibitor cocktail). The supernatants (SNs) of the lysates were estimated and equal amount of lysates were subjected to SDS-PAGE. After transfer to nitrocellulose membrane and subsequent blocking, the membranes were immunoblotted with their respective Abs against SOCS-3 or phosphorylated STAT-1, STAT-4, and ERK. Afterward, blot was stripped off to determine the expression of ERK and actin, and then developed using chemiluminescence kit (Lumigen, Inc., Southfield, MI, USA). Blots were scanned with the help of Image Quant LAS 4000 (GE Healthcare, Pittsburgh, PA, USA). Densitometric analysis was done using Scion image software.

### Cytokine ELISA

The cytokines in the culture SNs of AcrDC^post^ and AcrDC^pre^ were monitored by coating the ELISA plates overnight at 4°C with rat anti-mouse IFN-γ (2 µg/ml), IL-12 (1 µg/ml), TNF-α (2 µg/ml), IL-6 (1 µg/ml), and IL-10 (2 µg/ml) Abs in phosphate buffer (pH 9.6 and pH 6, 0.05 M). The unbound sites were blocked with BSA (1%) in PBS for 2 h at 37°C. The plates were washed with PBS-Tween-20 (0.05%) and culture SNs (50 µl) were added overnight at 4°C. Respective biotinylated Abs diluted in dilution buffer (1:1 solution of PBS-Tween-20 and blocking buffer) were added into plates and incubated for 2 h at 37°C. Thereafter, avidin-HRP (1:1,000) was added to plates and incubated at 37°C for 1 h. The usual procedures of washing and incubation were carried at each step. The color developed, due to H_2_O_2_-OPD substrate-chromogen, was stopped by H_2_SO_4_ (7%) and the plates were read at 492 nm. Serial dilutions of recombinant cytokines were used to prepare the standard curves to quantify the cytokines in the SNs.

### Evaluation of NF-κB by EMSA

AcrDC^post^ and AcrDC^pre^ were harvested on the sixth day, washed and treated with IFN-γ and LPS. After 30 min, nuclear extracts were prepared as mentioned elsewhere (6, 11). An equal amount of nuclear extract (10 µg) from each sample was incubated for 30 min/RT and labeled with P^32^-duplex oligonucleotides containing binding site for NF-κB in binding buffer. The DNA–protein complexes were resolved by electrophoresis on a native PAGE (7%). After electrophoresis, the gel was dried and exposed to screen at for 12 h/RT. The screen was scanned by phosphoimager (Fujifilm, Tokyo, Japan).

### Syngeneic T Cell Differentiation

Naïve T cells were purified by MACS, as per manufacturer’s instructions (BD Biosciences, San Diego, CA, USA). Briefly, after preparing single cell suspension, splenocytes were treated with CD4 T cell enrichment cocktail supplemented with biotinylated anti-CD44 and CD25 Abs. Later, same volume of streptavidin-magnetic beads was added and cells were isolated using BD IMagnet. The purity of naïve T cells was >97%, as ascertained by flow cytometry. Naïve CD4 T cells (3 × 10^5^/well, 24 well plate) were poured on anti-CD3 Ab (2 µg/ml) coated plate and cocultured with AcrDC^post^ and AcrDC^pre^ under Th1 (IL-12: 10 ng/ml, anti-IL-4 Ab: 5 µg/ml, IL-2: 100 U/ml), Th2 (IL-4: 5 ng/ml, anti-IFN-γ Ab: 5 µg/ml, anti-IL-12 Ab: 5 µg/ml) and Th17 (IL-6: 30 ng/ml, TGF-β: 5 ng/ml, anti-IL-4 Ab: 5 µg/ml, anti-IFN-γ Ab: 5 µg/ml, anti-IL-2 Ab: 5 µg/ml) polarization conditions.

### Allogeneic T Cell Differentiation

MACS purified naïve T cells (2 × 10^5^/well) of C57BL/6 mice were cocultured with AcrDC^post^ and AcrDC^pre^ of C3H/HeN in a 1:10 ratio (DC: T cells). After 3 days, the SNs were collected and estimated for IFN-γ and IL-17 by ELISA.

### *In Vivo* Polarization of T Cells by AcrDC^post^ and AcrDC^pre^

Mice (*n* = 8) were injected subcutaneously (s.c.) with OVA (100 µg) in alum (2 mg) dissolved in PBS (0.2 ml). Ten days later, AcrDC^post^ and AcrDC^pre^ incubated for 2 h with OVA (100 µg/ml) were adoptively transferred s.c. into OVA-primed mice. On the seventh day, the mice were sacrificed and LNs were isolated and single cell suspension was prepared. These cells were then cultured with OVA (100 µg/ml) for 48 h. Later, polarization of T cells was monitored by quantification of IFN-γ and IL-17 in the SNs by ELISA.

### Differentiation of OVA-Specific T Cells by AcrDC^post^ and AcrDC^pre^

Mice (*n* = 8) were sensitized s.c. with OVA (100 µg). After 7 days, T cells were purified from LNs by MACS. T cells were cocultured with OVA-pulsed AcrDC^post^ and AcrDC^pre^ at a ratio of 1:10 (DC: T cells) for 5 days. Later, IFN-γ and IL-17 were estimated in SNs by ELISA.

### AcrDC^post^ and AcrDC^pre^-Mediated Proliferation of T Cells

Mice (*n* = 8) were s.c. immunized with OVA in alum. After 10 days, T cells were isolated from LNs by MACS. The T cells were labeled with CFSE (1 µM) by incubating in PBS for 8 min at 37°C. The cells were washed 3× and cocultured with OVA-pulsed AcrDC^post^ and AcrDC^pre^ for 48 h. Proliferation was assayed by CFSE-dye dilution assay using a flowcytometer. The histogram data (stimulation index: SI) are represented as a bar diagram, SI = AcrDC^pre/post^cDCs. The flowcytometric analysis was done using BD FACS Diva software.

### Migration Assay for AcrDC^post^ and AcrDC^pre^

AcrDC^post^ and AcrDC^pre^ were labeled with either CFSE (2 µM) or efluor (2 µM) and adoptively transferred i.v. into mice (*n* = 8). After 2 days, animals were sacrificed. The presence of CFSE or efluor-positive cells in the spleen was monitored by FACSAria flowcytometer. The flowcytometric analysis was done using FACS Diva software.

### Quantitative PCR for TGF-β

RNA was isolated using Trizol reagent; according to the manufacturer’s instruction (Invitrogen, Carisbad, CA). Briefly, RNA was quantified by NanoDrop spectrophotometer. A260/A280 ratio of samples was in the range of 1.90–2.00. Intactness of RNA samples was determined by formaldehyde denaturing agarose gel electrophoresis. DNA contamination of RNA samples was removed by amplification grade DNase. Later, DNase activity was terminated by adding stop solution. Further, the samples were heated to 70°C for 10 min to inactivate DNase. Analysis was done by comparative Ct method. RT-qPCR and data analysis were done by Realplex Mastercycler (Eppendorf, Hamburg, Germany). The following primers were used for the quantification.

#### *Tgf*-*β*

FV 5′ TGACGTCACTGGAGTTGTACGG 3′
RV 5′ GGTTCATGTCATGGATGGTGC 3′

#### *β*-*Actin*

FV 5′-AGAGGGAAATCGTGCGTGAC-3′
RV 5′-CAATAGTGATGACCTGGCCGT-3′

Results are represented in the form of relative expression to actin m-RNA (fold change).

### Statistical Analysis

Statistical analysis was carried out using non-parametric Student’s *t*-test using GraphPad Prism (GraphPad Software, San Diego, CA, USA).

## Results

### Acr1 Exerts Distinct Effect on Encountering AcrDC^pre^ and AcrDC^post^

**Figure 1:**
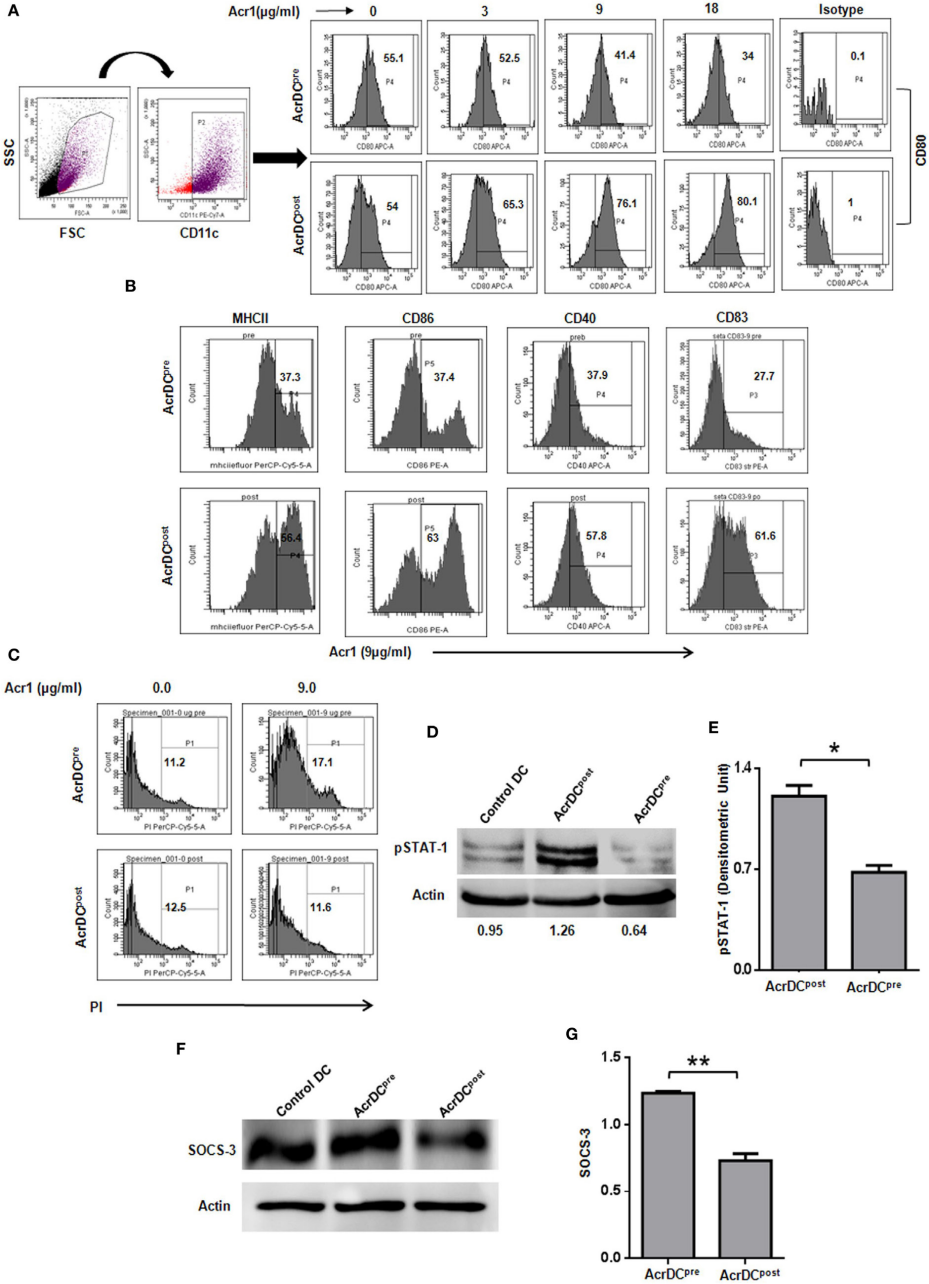
**Distinct role of alpha-crystallin protein (Acr1) in the maturation of BMDCs**. The BMDCs cultured with GM-CSF + IL-4 were exposed to Acr1 either during maturation (AcrDC^pre^) or after maturation (AcrDC^post^) and the expression on CD11c gated population was monitored by flow cytometry for the expression of **(A)** CD80 **(B)**; MHCII, CD86, CD40, CD83. **(C)** The AcrDC^pre^ and AcrDC^post^ were checked for viability by PI staining. The maturation status of AcrDC^pre^ and AcrDC^post^ was monitored by the phosphorylation of **(D,E)** STAT-1; **(F,G)** SOCS-3 by Western blotting and the densitometric analysis is expressed (mean ± SD) after normalizing with loading control actin. The values in the inset of flowcytometer histograms are percentage of cells. The results shown are representative of two to three independent experiments. *p < 0.05, **p < 0.01.

The optimum expression of MHC and costimulatory molecules is considered to be quite crucial in deciding the activation or anergy in T cells. Costimulatory molecules like CD80, CD86, CD40, and CD83 are regarded as maturation markers for DCs. Importantly, we noticed that Acr1 upregulates the CD80 molecule on AcrDC^post^ but downregulates CD80 on AcrDC^pre^. The change was noted in a dose-dependent manner (Figure 1A). Similarly, we observed the augmented expression of MHCII, CD86, CD40, and CD83 on AcrDC^post^, while downregulation in the levels of these molecules on AcrDC^pre^ (Figure 1B). Thus, substantiating the observed disparity in the expression of CD80 on AcrDC^pre^ and AcrDC^post^. Since, optimum expression of CD80 was induced at a dose of 9 µg/ml of Acr1, subsequently in all the experiments this concentration was used. In order to validate that the downregulation of costimulatory molecules in AcrDC^pre^ is not due to cell death, we stained AcrDC^pre^ and AcrDC^post^ with PI and no change in the viability of AcrDC^post^ and AcrDC^pre^ was observed (Figure 1C).

We next conducted experiments to identify the mechanism responsible for the distinct behavior of AcrDC^post^ and AcrDC^pre^. The STAT-1 is a critical transcription factor responsible for the activation and maturation of DCs (16). Therefore, we investigated the status of STAT-1 in AcrDC^post^ and AcrDC^pre^. Intriguingly, substantial enhancement in the level of STAT-1 phosphorylation in AcrDC^post^ but decline in AcrDC^pre^ was observed (Figures 1D,E). The SOCS-3 is a suppressor of cytokine signaling that negatively regulates STAT-1 mediated maturation of DCs (17–19). SOCS-3 has also been linked with the inhibition of CD4 T cells activation and proliferation (20). Intriguingly, we observed an inverse correlation between STAT-1 and SOCS-3 (Figures 1D–G). AcrDC^pre^ induced higher SOCS-3, whereas AcrDC^post^ displayed lower level. These results indicate that the mechanism responsible for the phenotypic and functional difference in AcrDC^post^ and AcrDC^pre^ is mediated through SOCS-3, which in turn regulates the STAT-1 mediated maturation of DCs.

**Figure 2:**
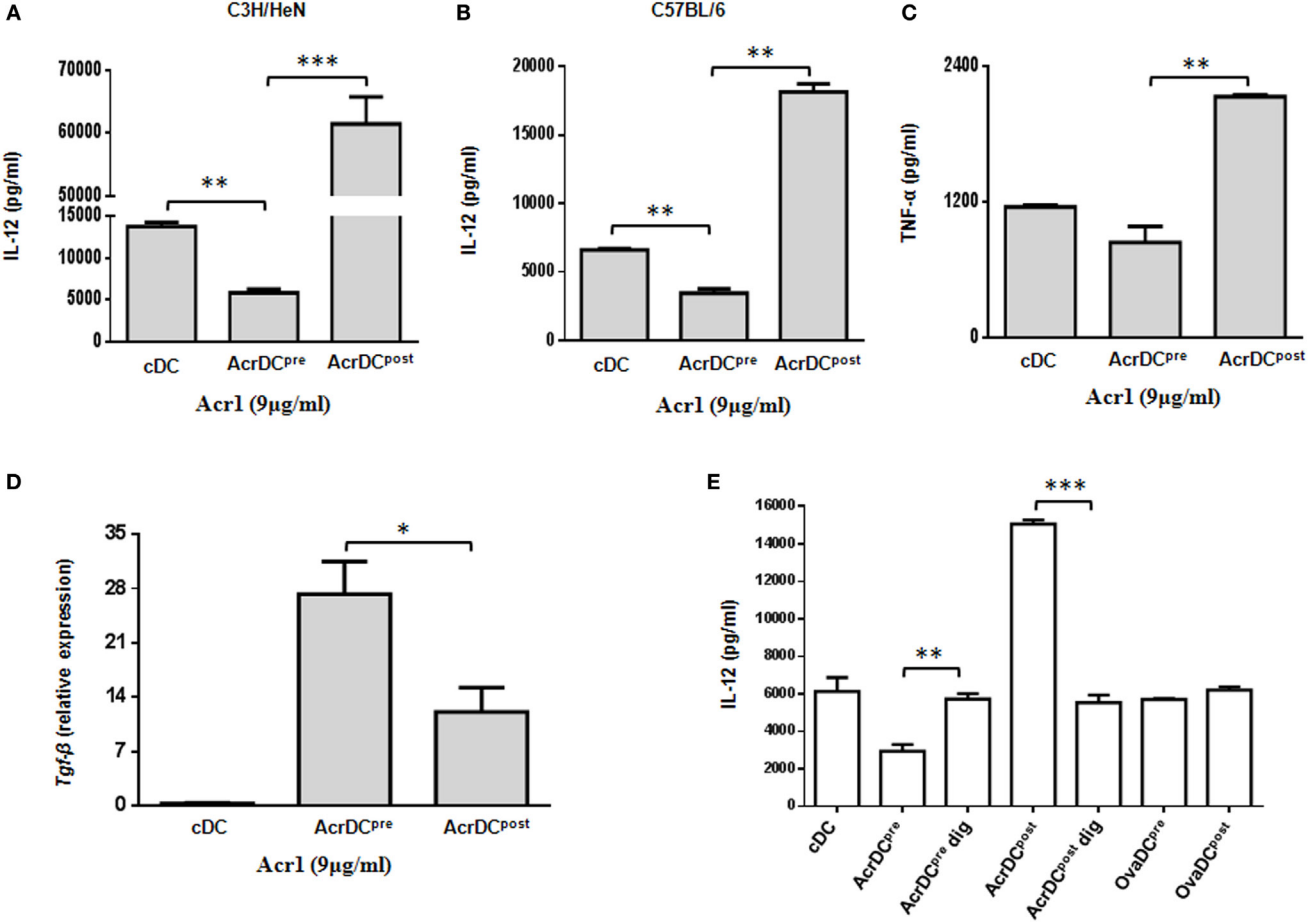
**Discrete secretion of proinflammatory cytokines by AcrDC^pre^ and AcrDC^post^**. The supernatants (SNs) collected from AcrDC^pre^ and AcrDC^post^ derived from **(A)** C3H/HeN; **(B)** C57BL/6 mice were estimated for the secretion of IL-12 by ELISA. Similarly, the SNs obtained from C3H/HeN AcrDC^pre^ and AcrDC^post^ were assessed for **(C)** TNF-α. **(D)** *Tgf-*β was monitored by RT-qPCR. The mRNA level was normalized with β-actin control and the data are depicted as relative expression. **(E)** The specificity of the activity of alpha-crystallin protein (Acr1) was established by culturing AcrDC^pre^ and AcrDC^post^ with Acr1 digested with proteinase K and estimating the release of IL-12 in the SNs. OVA is used as a control protein. The results expressed as mean ± SD are the representative of two to three independent experiments. AcrDC^pre^ dig and AcrDC^post^ dig: AcrDC^pre^ and AcrDC^post^ cultured with proteinase K digested Acr; OvaDC^pre^ and OvaDC^post^: DC^pre^ and DC^post^ cultured with OVA. *p < 0.05, **p < 0.01, ***p < 0.001.

The cytokines released by DCs are indispensable for the differentiation of T cells. We observed significant (*p* < 0.001) increase in the secretion of IL-12 by C3H/HeN AcrDC^post^, as compared to AcrDC^pre^ (Figures 2A,B). A similar trend in the secretion of IL-12 was noted in C57BL/6 strain, thereby illustrating that the impact of Acr1 is independent of any genetic diversity in the mice. IL-12 is a key regulator for the differentiation of Th1 cells. Like IL-12, a similar trend was noted in the case of TNF-α (Figure 2C). TNF-α plays a vital role in restricting the growth of *Mtb* (21). The results of AcrDC^pre^ are in consistent with our previous observation (6), we noticed substantial (*p* < 0.01) inhibition in the yield of IL-12 when BMCs were matured in the presence of Acr1. Further, as compared to AcrDC^post^, we observed significantly (*p* < 0.05) higher levels of TGF-β in AcrDC^pre^ (Figure 2D). Overall, these results indicate that Acr1 exposure inhibits the maturation of DC precursors during early stage of differentiation, whereas the post-maturation encounter of Acr1 potentiates DCs development.

To establish the specificity of Acr1 and its observed effect is not due to any artifact, we digested Acr1 with proteinase K and cultured it with AcrDC^pre^ and AcrDC^post^ and studied the secretion of IL-12. Interestingly, no change in the release of IL-12 was observed by AcrDC^pre^ and AcrDC^post^ cultured with proteinase K digested Acr1, but undigested Acr1 retained its modulatory function (Figure 2E), explicitly confirming the specificity of Acr1 in modulating DCs functionality. No change was noticed when AcrDC^pre^ and AcrDC^post^ cultures were supplemented with OVA in lieu of Acr1, thereby further validating the specificity of Acr1.

### Differential Production of IL-12 by AcrDC^pre^ and AcrDC^post^ Is Mediated through ERK Signaling

**Figure 3:**
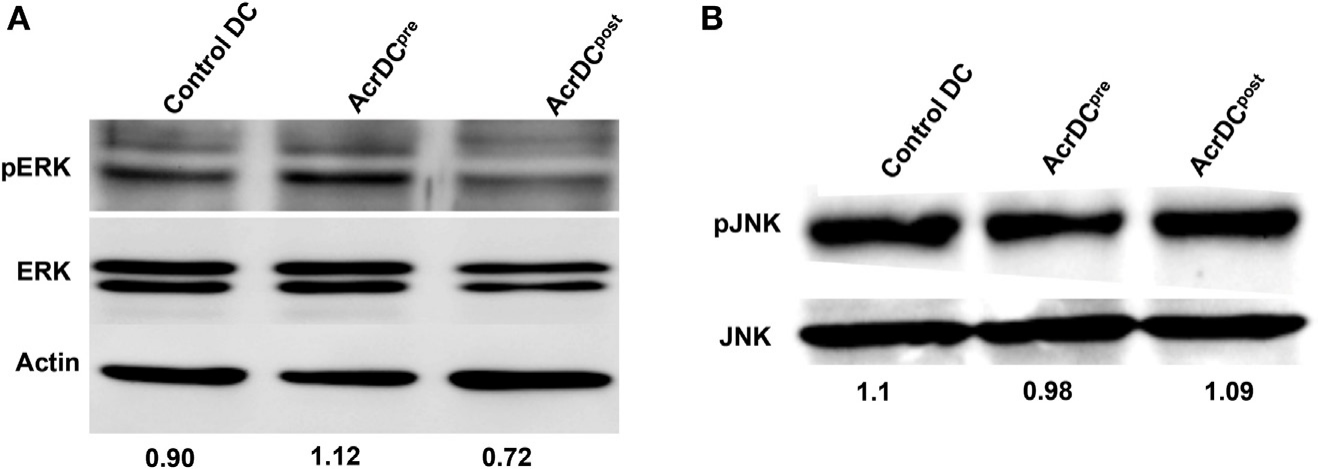
**Difference in the yield of IL-12 by AcrDC^pre^ and AcrDC^post^ is mediated through ERK pathway**. **(A,B)** The variance in the levels of IL-12 by AcrDC^pre^ and AcrDC^post^ is mediated through ERK but not JNK pathway, as evidenced by the modulation in the phosphorylation of ERK and JNK monitored by Western blotting. Western blots are the representative of two to three independent experiments.

Next, we elucidated the mechanism by which Acr1 elicited the IL-12 production. The MAPK pathway is critical in regulating the expression of IL-12. Therefore, we investigated the status of ERK and JNK activation. We observed augmented phosphorylation of ERK in AcrDC^pre^ (Figure 3A). In contrast, suppression was noted in AcrDC^post^. No change was discernible in JNK phosphorylation (Figure 3B), indicating that Acr1 mediates the regulation of IL-12 through the ERK pathway.

### The Upregulation in the Expression of CCR7 on AcrDC^post^ Correlates with Their Greater Migratory Potential and Ability to Help T cells

**Figure 4:**
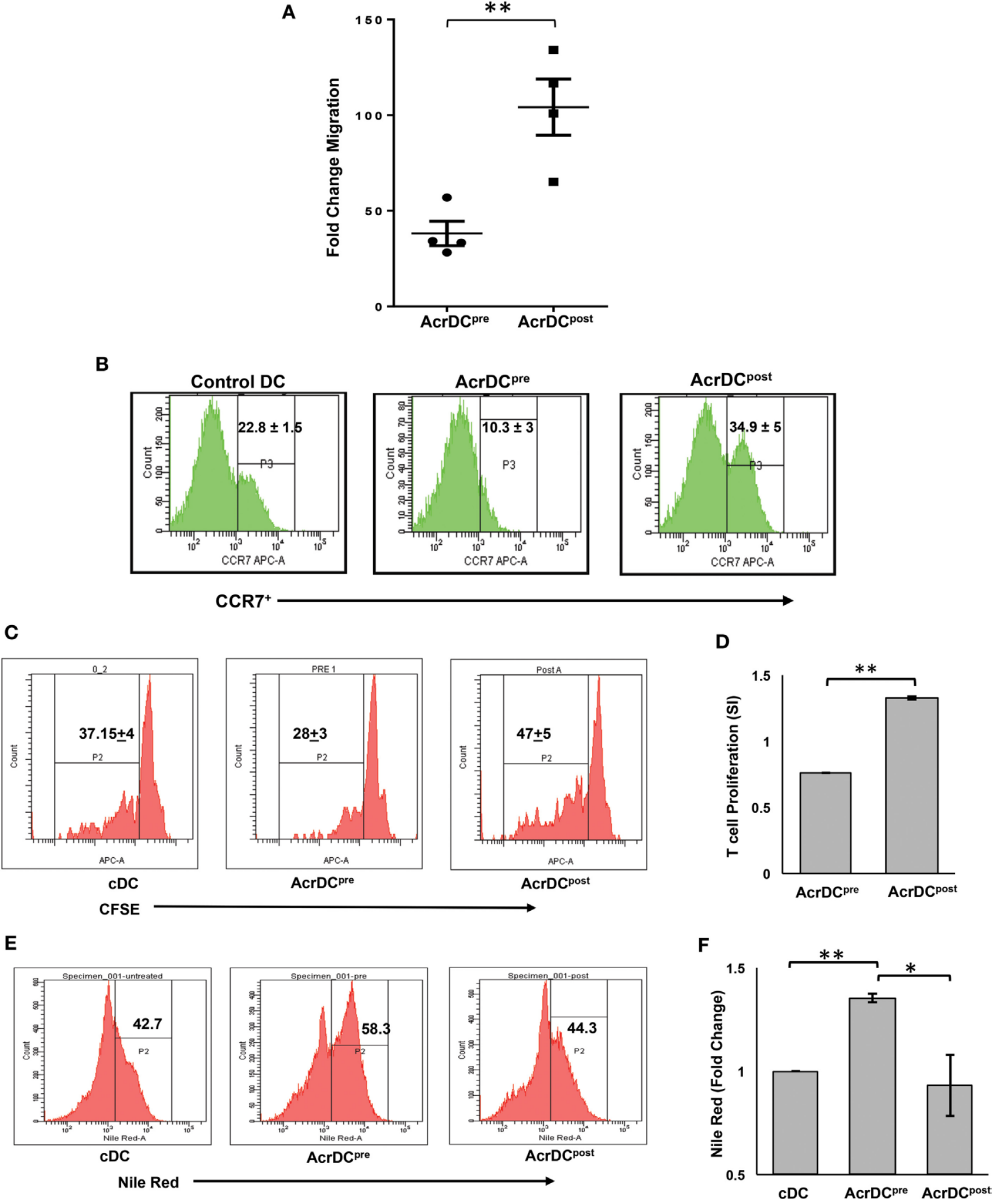
**AcrDC^pre^ and AcrDC^post^ exhibited disparity in their migratory preferences and activation of T cells**. **(A)** The adoptive transfer of CFSE-labeled AcrDC^pre^ and AcrDC^post^ in the mice established that AcrDC^pre^ migration to spleen was significantly lesser than AcrDC^post^. Each dot indicates single animal. The fold change in the migration was calculated by normalizing with untreated dendritic cells (DCs). **(B)** AcrDC^pre^ showed downregulation in the expression of migratory chemokine CCR7. In contrast, AcrDC^post^ displayed upregulation of CCR7, as monitored by flow cytometry. **(C,D)** T cells isolated from OVA immunized mice labeled with CFSE were cocultured with AcrDC^pre^ and AcrDC^post^ for 48 h. The proliferation was enumerated by flow cytometry and the data are expressed as histograms. The histogram data (stimulation index: SI) are represented as bar diagram, SI = AcrDC^pre/post^/cDCs. **(E,F)** The AcrDC^pre^ and AcrDC^post^ were stained with Nile red dye to estimate the accumulation of lipids and data are expressed as flow cytometry histograms and bar diagram. The figures in the inset of histograms signify percent positive cells. The data represented as mean ± SD are of two to three independent experiments (*n* = 4/group). *p < 0.05, **p < 0.01.

The trafficking of DCs is critical for the activation of T cells. To observe this phenomenon, we adoptively transferred CFSE-labeled AcrDC^post^ and AcrDC^pre^ into mice and monitored their migration to the spleen. We noted a remarkable increase (*p* < 0.01) in the movement of AcrDC^post^, as compared to AcrDC^pre^ (Figure 4A). Interestingly, we observed higher expression of CCR7 on AcrDC^post^ that may explain their greater trafficking capability (Figure 4B). Furthermore, we investigated whether AcrDC^post^ exhibited enhancement in their potential to activate T cells. We observed that AcrDC^post^ showed greater ability to help T cells, as evidenced by improved (*p* < 0.01) proliferation of T cells (Figures 4C,D). In contrast, AcrDC^pre^ significantly (*p* < 0.01) inhibited the proliferation of T cells, as compared to control DCs (cDCs).

We next examined why AcrDC^pre^ expressed inhibitory activity. Recently, it has been reported that DCs with accumulated lipids exhibit dysfunction in their activity (22). This prompted us to check whether the malfunctioning of AcrDC^pre^ can be attributed due to their lipid content. We observed that AcrDC^pre^ accumulated a substantially larger amount of lipids than AcrDC^post^ (*p* < 0.05) or cDCs (*p* < 0.01) (Figures 4E,F; Figure S1 in Supplementary Material). Consequently, higher lipid content may be one of the reasons for the suppressive nature of AcrDC^pre^.

### AcrDC^post^ Promotes but AcrDC^pre^ Inhibits the Differentiation of Th1 Cells and Th17 Cells

**Figure 5:**
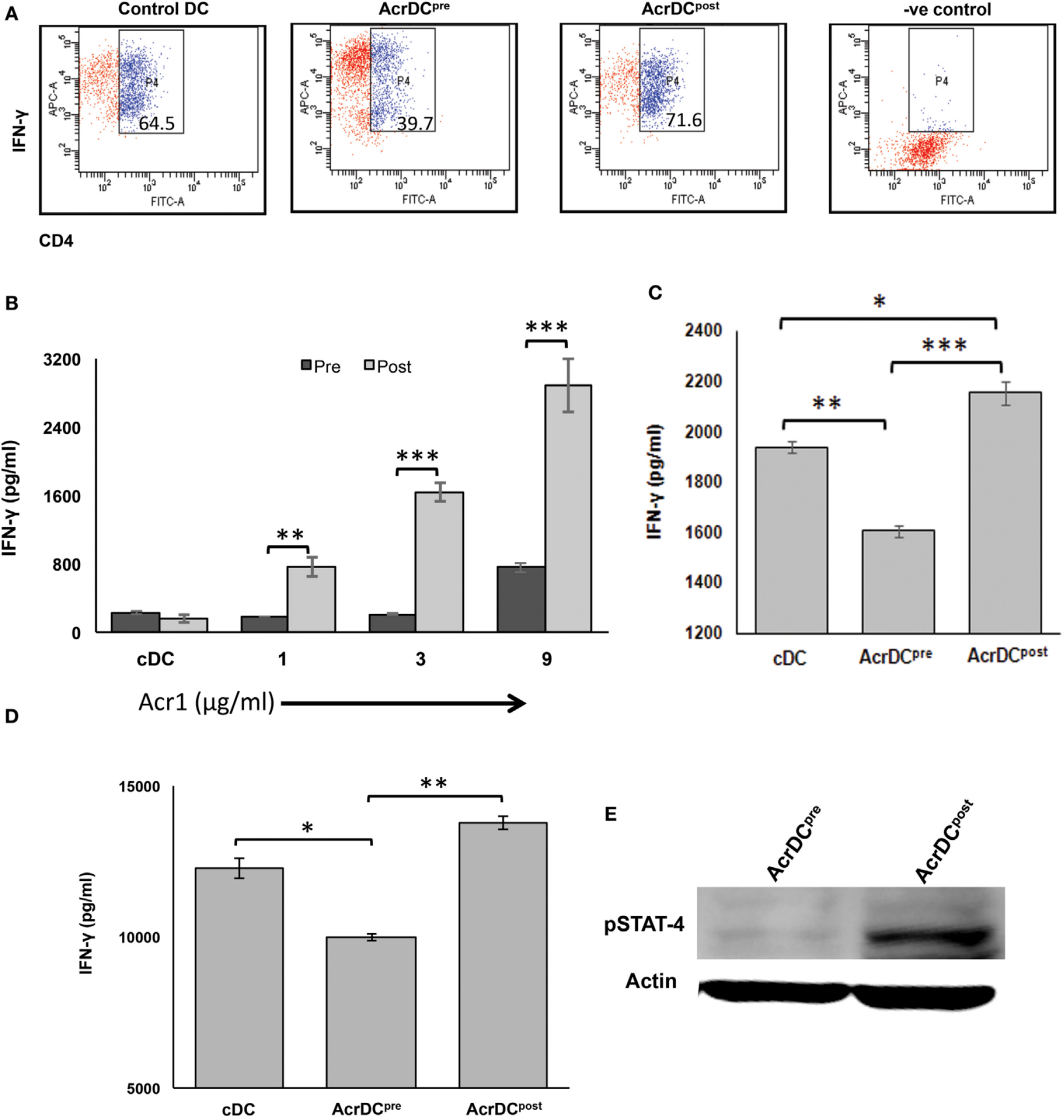
**AcrDC^pre^ inhibits, while AcrDC^post^ supports the differentiation of naïve T cells to Th1 cells by modulating the expression of STAT-4**. **(A)** Anti-CD3 Ab-stimulated naïve CD4 T cells were cocultured with AcrDC^pre^ and AcrDC^post^ under Th1 cell differentiation conditions (IL-12 + anti-IL4 Ab), and monitored for the intracellular expression of IFN-γ by flow cytometry. AcrDC^post^ facilitates, in contrast AcrDC^pre^ suppresses the secretion of IFN-γ when cocultured with **(B)** allogeneic (C57BL/6) naïve T cells; **(C)** CD4 T cells isolated from OVA immunized animals were cocultured with AcrDC^pre^ and AcrDC^post^ for 5 days. Later, IFN-γ was estimated in the supernatants (SNs) by ELISA. **(D)** OVA-pulsed AcrDC^pre^ and AcrDC^post^ were adoptively transferred in the OVA-primed mice. After 7 days, LN cells were isolated and cultured with OVA for 48 h. The IFN-γ was estimated in the culture SNs by ELISA. The flow cytometry data in the inset illustrate percent positive cells and bar diagram of ELISA (mean ± SD) are representative of two to three independent experiments. *p < 0.05, **p < 0.01; ***p < 0.001. **(E)** Phosphorylation status of STAT-4 in AcrDC^pre^ and AcrDC^post^ was monitored by Western blotting. Actin was used as a loading control. The blot shown is an illustrative of three individual experiments.

We noticed the discrete role of AcrDC^post^ and AcrDC^pre^ in modulating the secretion of IL-12 (Figure 2). DCs play a cardinal role in the activation and differentiation of naïve CD4 T cells. IL-12 is a differentiation factor for Th1 cells. Therefore, it was worth to monitor the influence of AcrDC^post^ and AcrDC^pre^ in the generation of Th1 cells. AcrDC^post^ and AcrDC^pre^ were cultured with either anti-CD3 Ab stimulated naïve CD4 T cells under Th1 differentiation conditions (Figure 5A) or allogeneic naïve CD4 T cells (Figure 5B) or antigen specific T cells (Figure 5C). Intriguingly, AcrDC^post^ promoted but AcrDC^pre^ retarded the differentiation of Th1 cells, as evidenced by higher expression of IFN-γ by AcrDC^post^. This change was detected in both antigen-specific and allogeneic CD4 T cells (Figures 5A–C). Furthermore, we also adoptively transferred OVA-pulsed AcrDC^post^ and AcrDC^pre^ into the mice that were primed with OVA. Like the *in vitro* results, it was noticed *in vivo*, as well that AcrDC^post^ potentiated while AcrDC^pre^ inhibited the differentiation of Th1 cells (Figure 5D).

STAT-4 activation is responsible for the IFN-γ-inducible genes (23). Therefore, we further validated AcrDC^post^ mediated differentiation of Th1 cells by examining the activation of STAT-4. It was noticed that AcrDC^post^ showed higher phosphorylation of STAT-4 (Figure 5E). Contrary to this, AcrDC^pre^ exhibited substantial decline in STAT-4. This is, therefore, indicative of the role of STAT-4 pathway in promoting the differentiation of Th1 cells by AcrDC^post^.

**Figure 6:**
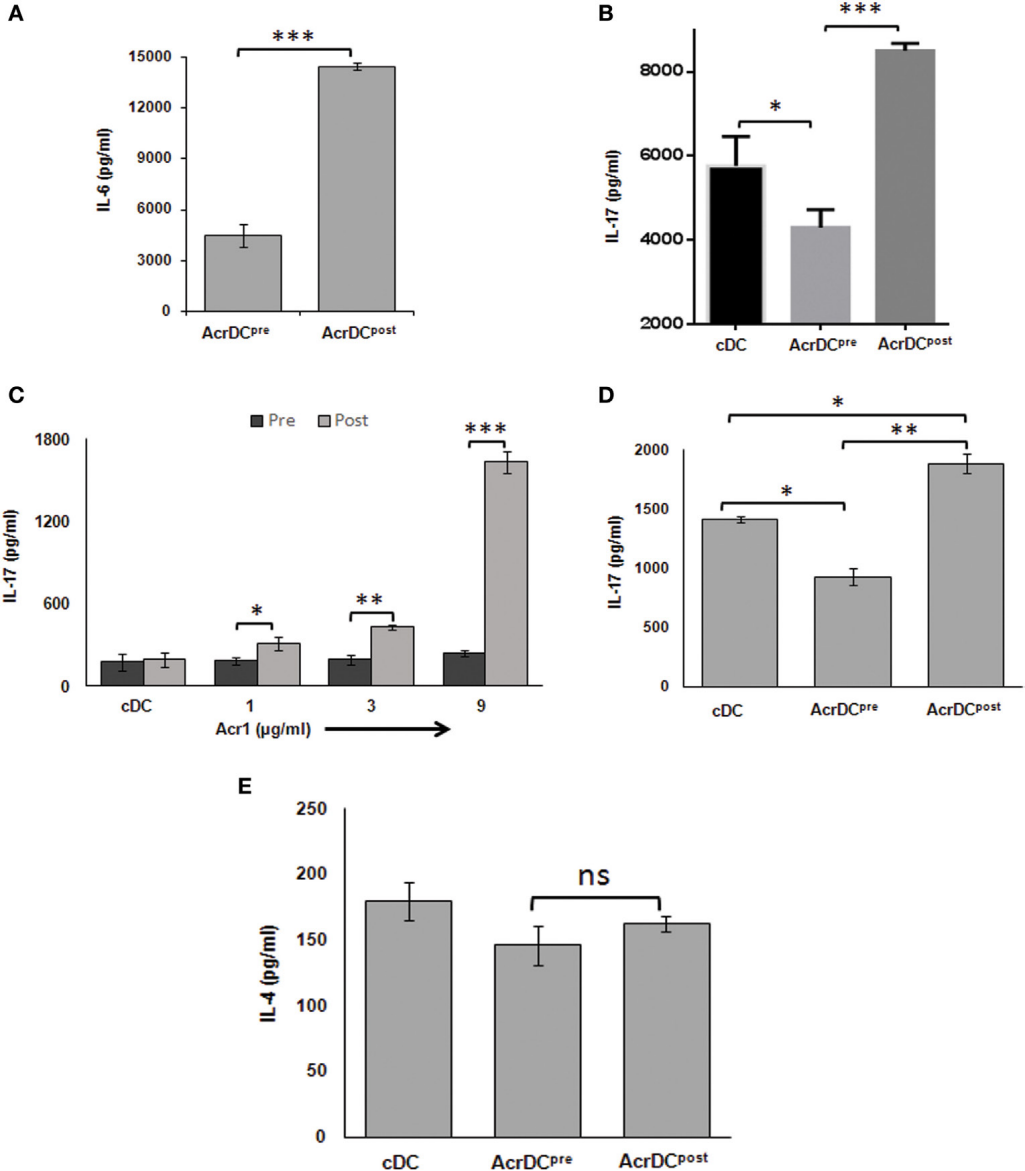
**AcrDC^pre^ impedes but AcrDC^post^ promotes the differentiation of Th17 cells**. **(A)** IL-6 was estimated in the supernatants (SNs) of AcrDC^post^ and AcrDC^pre^. IL-17 was monitored in SNs of AcrDC^post^ and AcrDC^pre^ cultured with **(B)** anti-CD3 Ab stimulated naïve T cells under Th17 differentiation conditions (IL-6 ± TGF-β); **(C)** allogeneic naïve T cells (C57BL/6); **(D)** OVA-specific T cells. **(E)** AcrDC^pre^ and AcrDC^post^ showed no difference in the yield of IL-4 when cocultured with OVA-specific T cells. The cytokines were measured by ELISA. The data (mean ± SD) expressed as bar diagram are from two to three independent experiments. *p < 0.05, **p < 0.01; ***p < 0.001.

Th17 cells play a dual role not only in protection but also in the pathogenesis of the diseases, and TGF-β and IL-6 cytokines are necessary for their differentiation (24). We observed disparate production of TGF-β (Figure 2D) and IL-6 (Figure 6A) by AcrDC^post^ and AcrDC^pre^. Consequently, we were curious to know whether AcrDC^post^ and AcrDC^pre^ can distinctly regulate Th17 cells. AcrDC^post^ and AcrDC^pre^ were cultured with anti-CD3 Ab stimulated naïve CD4 T cells under Th17 differentiation conditions (Figure 6B), allogeneic naïve CD4 T cells (Figure 6C), and antigen-specific T cells (Figure 6D). Like Th1 cells, AcrDC^post^ facilitated, whereas AcrDC^pre^ impeded the differentiation of Th17 cells (Figures 6B–D). No change was observed in Th2 cells differentiation by AcrDC^post^ and AcrDC^pre^ (Figure 6E).

### AcrDC^post^ Controls but AcrDC^pre^ Fails to Restrict the Intracellular Growth of *Mtb*

**Figure 7:**
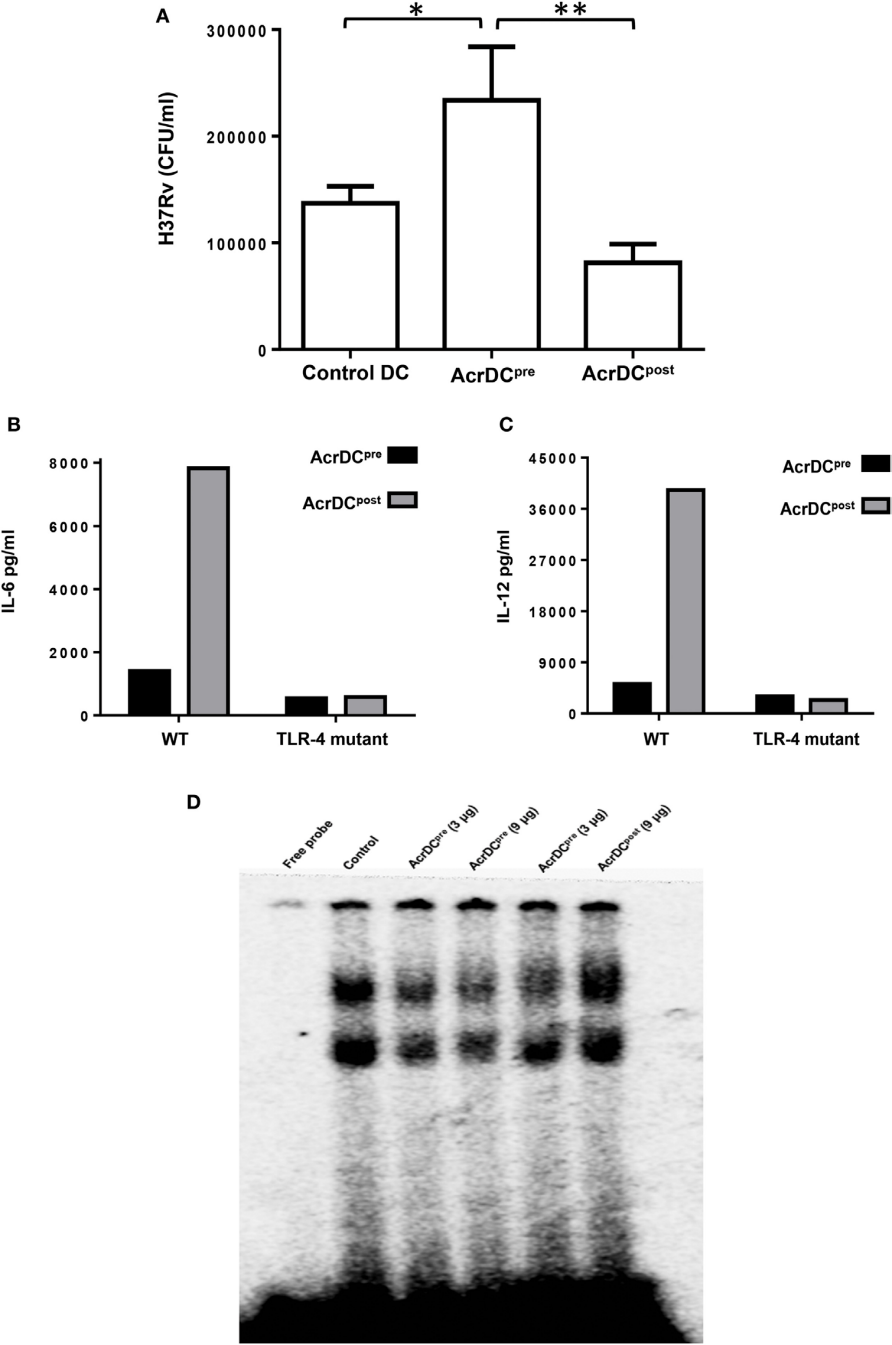
**AcrDC^post^ but not AcrDC^pre^ restricted the intracellular growth of *Mycobacterium tuberculosis* (*Mtb*)**. **(A)** AcrDC^pre^ and Acr1DC^post^ were infected with *Mtb* H37Rv for 24 h. Later, the infected dendritic cells (DCs) were lysed and plated on 7H11 agar plates and bacterial load was counted after 20 days and expressed as CFU/ml. **(B)** Dissimilar response in AcrDC^pre^ and AcrDC^post^ is mediated by TLR-4. AcrDC^pre^ and AcrDC^post^ were generated from WT and TLR-4 mutant mice and estimated the secretion of **(B)** IL-6; **(C)** IL-12 in the supernatants by ELISA. **(D)** AcrDC^pre^ prevented but AcrDC^post^ promoted the nuclear translocation of NF-κB. Nuclear extracts of AcrDC^pre^ and AcrDC^post^ cultured with the indicated concentrations of alpha-crystallin protein (Acr1) were prepared and checked for NF-κB binding to DNA by EMSA. Data (mean ± SD) are representative of two to three independent experiments. *p < 0.05, **p < 0.01; ***p < 0.001.

*Mycobacterium tuberculosis* is an extremely successful pathogen because it can thwart its host’s immune system and thrives in dormant state in a hostile environment of one-third of world’s population. Acr1 is predominantly expressed by *Mtb* during latency (8, 25). The current study illustrates that Acr1 alters the function of AcrDC^post^ and AcrDC^pre^, so we wanted to investigate their abilities to restrict the growth of *Mtb*. Importantly, as compared to AcrDC^pre^, AcrDC^post^ notably (*p* < 0.01) constrained the survival of *Mtb* (Figure 7A). However, *Mtb* showed unobstructed growth in AcrDC^pre^; which also supports our earlier study (6). These results suggest the dual role of Acr1 in controlling the stage-specific activity of DCs.

### Differential Regulation of AcrDC^pre^ and AcrDC^post^ by Acr1 Is Mediated through TLR-4

It is reported that Acr1 binds to TLR-4 (15). Therefore, we thought it would be prudent to evaluate the role of TLR-4 in Acr1 mediated modulation of DCs. Interestingly, AcrDC^post^ derived from TLR-4 mutant mice (C3H/HeJ) showed abrogation in the secretion of IL-6 and IL-12 (Figures 7B,C). Wild-type mice (C3H/HeN), on the other hand, exhibited augmented production of these cytokines. These results indicate that the modulatory activity of Acr1 on DCs may be dependent on the extent of TLR-4 signaling. Triggering through TLR-4 is reported to induce both activation and inhibition in the function of DCs (19). Consequently, this study validates our results.

### The Modulatory Function of AcrDC^post^ and AcrDC^pre^ Is Regulated through NF-κB Activation

NF-κB plays a key role in orchestrating the immune response upon microbial infection. NF-κB translocation to the nucleus leads to upregulation of various cytokines, chemokines, and inflammatory mediators, which are required for the migration of immune cells to the site of infection. This study suggests the stimulatory phenotype of AcrDC^post^ (CD80^hi^, CD86^hi^, CD40^hi^, CD83^hi^, MHCII^hi^, IL-6^hi^, IL-12^hi^, TNF-α^hi^, TGF-β^lo^) and suppressive phenotype of AcrDC^pre^(CD80^lo^, CD86^lo^, CD40^lo^, CD83^lo^, MHCII^lo^, IL-6^lo^, IL-12^lo^, TNF-α^lo^, TGF-β^hi^). Therefore, we investigated the NF-κB activation in AcrDC^post^ and AcrDC^pre^. AcrDC^post^ exhibited significant enhancement but AcrDC^pre^ showed impediment in the nuclear translocation of NF-κB (Figure 7D), indicating that the differential responses detected in AcrDC^pre^ and AcrDC^post^ related to costimulatory molecules, cytokines, and proliferation are coordinated through NF-κB signaling.

## Discussion

One-third of world’s population is estimated to have latent tuberculosis infection. These individual serves as a reservoir for the pathogen and therefore pose a serious threat of spreading tuberculosis and providing a major impediment in the complete eradication of the disease. Although the host employs its immuno-artillery to combat *Mtb*, the pathogen not only diffuses the attack and survives but also releases factors to overpower the immunity. Acr1 is one of the predominantly expressed proteins during the dormancy. Its role as a virulence factor that impairs the differentiation and function of DCs has been surfaced (6, 26). In contrast, due to its immunodominant nature, it can be a potential vaccine candidate (27).

We have demonstrated that during differentiation if DCs encounter Acr1, it leads to the impairment in their maturation and functionality, resulting in the generation of tolerogenic phenotype (6). The *Mtb* showed better survival in such DCs. In contrast, a recent study suggested an adjuvant property of Acr1 treated DCs, in protection against tumor. Consequently, we thought to evaluate the impact of Acr1 on the differentiated DCs. To do so, we stimulated differentiated DCs with Acr1 (AcrDC^post^) and following major findings have emerged in context with the promotion of the maturation of DCs as evidenced by augmented (1) expression of MHCII and costimulatory molecules; (2) release of proinflammatory cytokines; (3) CCR7 display and migratory capacity; (4) potential to restrict the growth of *Mtb*; (5) ability to proliferate and differentiate T cells; (6) mechanism involved in this phenomenon was through TLR-4-mediated activation of STAT-1, ERK, and NF-κB; and inhibition in the SOCS-3 pathways (Figure S2 in Supplementary Material).

The delivery of signals through costimulatory molecules like CD80, CD86, CD83, and CD40 by DCs in conjunction with MHC–peptide complex is crucial for the activation of T cells. Contrary to this, downregulation of costimulatory molecules can lead to anergy in T cells. Costimulatory molecules are widely considered as maturation and differentiation markers for DCs and other APCs (28, 29). CD83 is an established maturation marker for DCs (30). DCs deficient in CD83 resulted in lesser T cell proliferation with impaired IFN-γ secretion (30). CD83 induces the expression of CD80, CD86, and MHCII molecules (31–33), thus boosting the overall functionality of DCs. Recruitment of CD86 and CD80 to lipid rafts on DCs is essential for optimal T cell activation (34). Similarly, we noted that AcrDC^post^ but not AcrDC^pre^ could optimally activate T cells. We postulate that the diminished activity of AcrDC^pre^ to activate T cells may be due to their reduced migratory capacity, as evidenced by the decreased display of CCR7. Furthermore, downregulation of the expression of costimulatory molecules and increased lipid content may be one of the reasons for the impairment of the function of AcrDC^pre^ to activate T cells to proliferate and secrete cytokines (22).

AcrDC^post^ chiefly promoted the proliferation and activation of naïve CD4 T cells toward Th1 cells and Th17 cells. In contrast, inhibition in the differentiation capacity of AcrDC^pre^ was observed. This may be due to the fact that AcrDC^post^ showed enhanced production of IL-12, IL-6, and TGF-β, which are the important factors for the differentiation of Th1 cells and Th17 cells, respectively (35, 36). Th1 cells and Th17 cells play a fundamental role in controlling the survival of intracellular pathogens like *Mtb*, leishmania, malaria, HIV, etc. (5, 37, 38). We were interested to know whether the modulation in the function of AcrDC^post^ and AcrDC^pre^ was explicitly due to Acr1. To establish this, we digested Acr1 with proteinase K and observed abrogation in the function of AcrDC^post^, as evidenced by inhibition in the secretion of IL-12. In addition, we used OVA, a non-Acr1 antigen and detected no discernible change in the yield of IL-12 by either AcrDC^post^ or AcrDC^pre^. Consequently, we established that the observed results are specifically due to Acr1.

The IL-12-mediated expression of CCR7 is critical for DCs migration to secondary lymphoid organs during *Mtb* infection (39). Similarly, we observed augmented production of IL-12 and higher expression of CCR7 in the case of AcrDC^post^, which explains the greater migratory potential of AcrDC^post^. These results corroborated with earlier findings (39).

One of the important features of APCs such as macrophages and DCs is to phagocytose and eliminate the pathogen. If the APCs fail to kill the phagocytosed bacteria, it will lead to bacterial survival and disease progression. Therefore, it was important to monitor whether the AcrDC^post^ were able to confine the intracellular growth and persistence of *Mtb*. AcrDC^post^ revealed significantly greater restriction of *Mtb*, as compared to AcrDC^pre^, indicating that Acr1 enhanced the killing potential of AcrDC^post^.

This study suggests the stimulatory as well as inhibitory role of Acr1, depending on the different stages of maturation of DCs. Hence, we propose here a mechanism responsible for this discrepancy. STAT-1 phosphorylation is an important indicator for the maturation and activation of DCs (16). Accordingly, we observed that AcrDC^post^ exhibited remarkably higher expression of pSTAT-1 but AcrDC^pre^ showed sizable decline in the activation of STAT-1. Further, we validated the data by examining the expression of the SOCS-3, which is a negative regulator of DCs maturation (16). We observed sufficient decrease in the SOCS-3 level, which may be responsible for the activation of AcrDC^post^. Furthermore, the stimulatory function of AcrDC^post^ was confirmed by the enhancement in the nuclear translocation of NF-κB. NF-κB contributes indispensably to the maturation of DCs and its blockage abrogates the process (40). ERK is responsible for regulating the secretion of IL-12 (41). DCs obtained from *Erk1* knockout mice showed profound production of IL-12 (42), illustrating that IL-12 production is negatively regulated by the ERK pathway (41). We observed that the phosphorylation of ERK was significantly higher in AcrDC^pre^, as compared to AcrDC^post^. Like ERK, JNK activation is also known to regulate IL-12 expression; however, no change in the phosphorylation of JNK was seen. This suggests that Acr1 regulates the production of IL-12 through the ERK pathway.

Recently, it has been demonstrated that Acr1 binds to TLR-4 and triggers the immuno-adjuvanting effect of DCs against tumors. Furthermore, Acr1 was shown to be a potent TLR-4 agonist that can enhance both DC activation and Th1 polarization through the MyD88 and TRIF signaling pathways (15). Acr1 activates the tyrosine phosphorylation of TLR-4, thus enhancing the MyD88 and TRIF-mediated signaling cascade. MyD88-dependent pathway is critical for the production of proinflammatory cytokines, while TRIF pathway is required for the expression of costimulatory molecules. Therefore, we thought it would be judicious to monitor the influence of Acr1 on DCs derived from TLR-4 mutant mice. Consequently, we noted that Acr1 failed to evoke the release of IL-12 and IL-6 by AcrDC^post^ and AcrDC^pre^ derived from TLR-4 mutant mice. In contrast, AcrDC^post^ from wild-type animals revealed substantially higher yield of IL-12 and IL-6. Thus, it may be inferred that *Mtb* employs its Acr1 to deliver signals through TLR-4 in order to modulate the function and phenotype of DCs. The inhibitory activity of Acr1 on AcrDC^pre^ through TLR-4 is in accordance with the study depicting that the engagement of DC precursors by pathogens or their TLRs ligand lead to the obstruction in the differentiation of DCs (43, 44). In contrast, Acr1 stimulation of AcrDC^post^ through TLR-4 promotes their maturation and activation capabilities (19, 40, 45). We suggest that this may be one of the reasons for the dual role of Acr1 on DCs.

Dendritic cell lineage comprises of cells at various stages of maturation and differentiation. Consequently, when either of these DCs encounter Acr1, it may lead to their stimulation or tolerization, which ultimately influences the activation or suppression of T cells. Earlier, it has been suggested that *Mtb* can reach and hide within bone marrow. It implies that *Mtb* might utilize its Acr1 to interfere with the proper differentiation of DCs precursor, which may be considered as one of its immune evasion strategy (46). On the other hand, at the site of infection, the host overpowers the bug by priming the differentiated DCs with Acr1 to activate the cells of the immune system and foil *Mtb* attack. This is also supported by the previously published study demonstrating immunoprotective role of recombinant BCG expressing Acr1. Thus, this study suggests that besides Acr1 utility in TB, its role as an immunotherapeutic agent may also be of immense importance in potentiating the activity of DCs in cancer therapy. In contrast, inhibition of the function of DCs can be a viable strategy for treating autoimmune diseases.

## Ethics Statement

Mice were procured from the animal house facility of Institute of Microbial Technology, Chandigarh, India. Institutional Animal Ethics Committee (IAEC) and regulatory guideline issued by the Committee for the Purpose of Supervision of Experiments on Animals (No. 55/1999/CPCSEA), Ministry of Environment and forest, Government of India.

## Author Contributions

The origin of concept was done by JA and designing of experiments by JA and MA. The experimental procedures and data analysis were conducted by MA, M Aqdas, SN, KS, NK, and JS. The manuscript was written by MA and JA.

## Conflict of Interest Statement

The authors declare that the research was conducted in the absence of any commercial or financial relationships that could be construed as a potential conflict of interest.

## Supplementary Material

Figure S1 **The AcrDC^pre^ and AcrDC^post^ display distinct lipid levels**. The AcrDC^pre^ and AcrDC^post^ were stained with Nile red dye and the expression of lipids was observed under confocal microscopy (magnification: 60×). The data are the representative of two independent experiments.

Figure S2 **Alpha-crystallin protein (Acr1) differentially regulates the functionality of DCs**. **(A)** During the differentiation, Acr1 abrogates the activation and maturation of DCs. In AcrDC^pre^, Acr1 suppresses the maturation of DCs by upregulating SOCS-3 which in turn inhibits activation of STAT-1. Moreover in AcrDC^pre^ the phosphorylation of ERK is promoted while the translocation of NF-κB is hampered, leading to suppressed expression of proinflmmatory cytokines. **(B)** In contrast, Acr1 interaction with TLR-4 after maturation leads to SOCS-3 downregulation and enhanced activation of STAT-1. Furthermore, it also leads to downregulation of phosphorylation of ERK and facilitates the nuclear translocation of NF-κB. The activation of NF-κB leads to the upregulation of proinflammatory cytokines.
