# Supplementary figures and images for: Diametric Role of the Latency-Associated Protein Acr1 of Mycobacterium tuberculosis in Modulating the Functionality of Pre- and Post-maturational Stages of Dendritic Cells
Source: Front Immunol. 2017 May 30;8:624. doi: 10.3389/fimmu.2017.00624 (PMC5447689; doi:10.3389/fimmu.2017.00624)

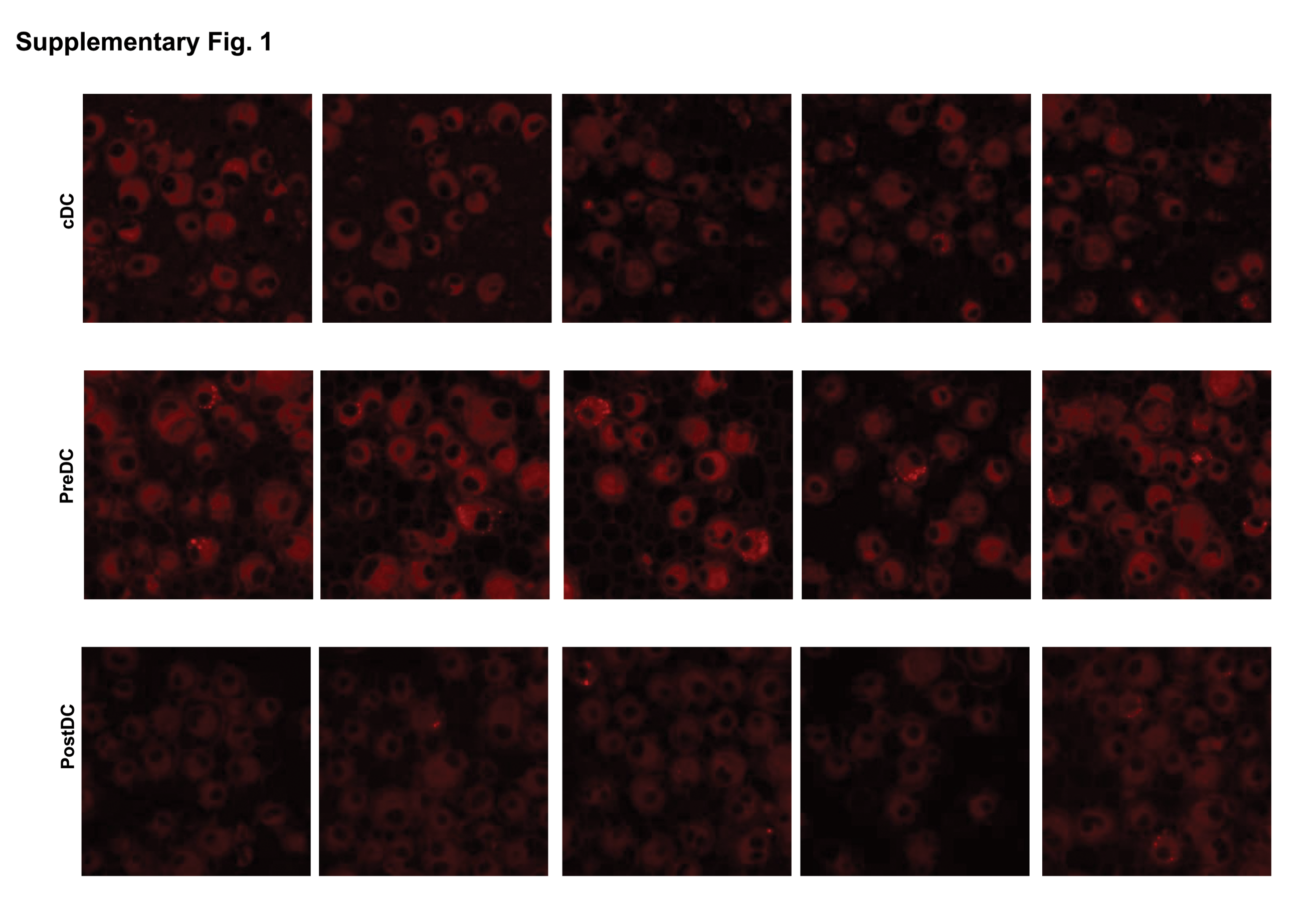

Supplement: Figure S1 — The AcrDCpre and AcrDCpost display distinct lipid levels. The AcrDCpre and AcrDCpost were stained with Nile red dye and the expression of lipids was observed under confocal microscopy (magnification: 60×). The data are the representative of two independent experiments. [file Image_1.tif]

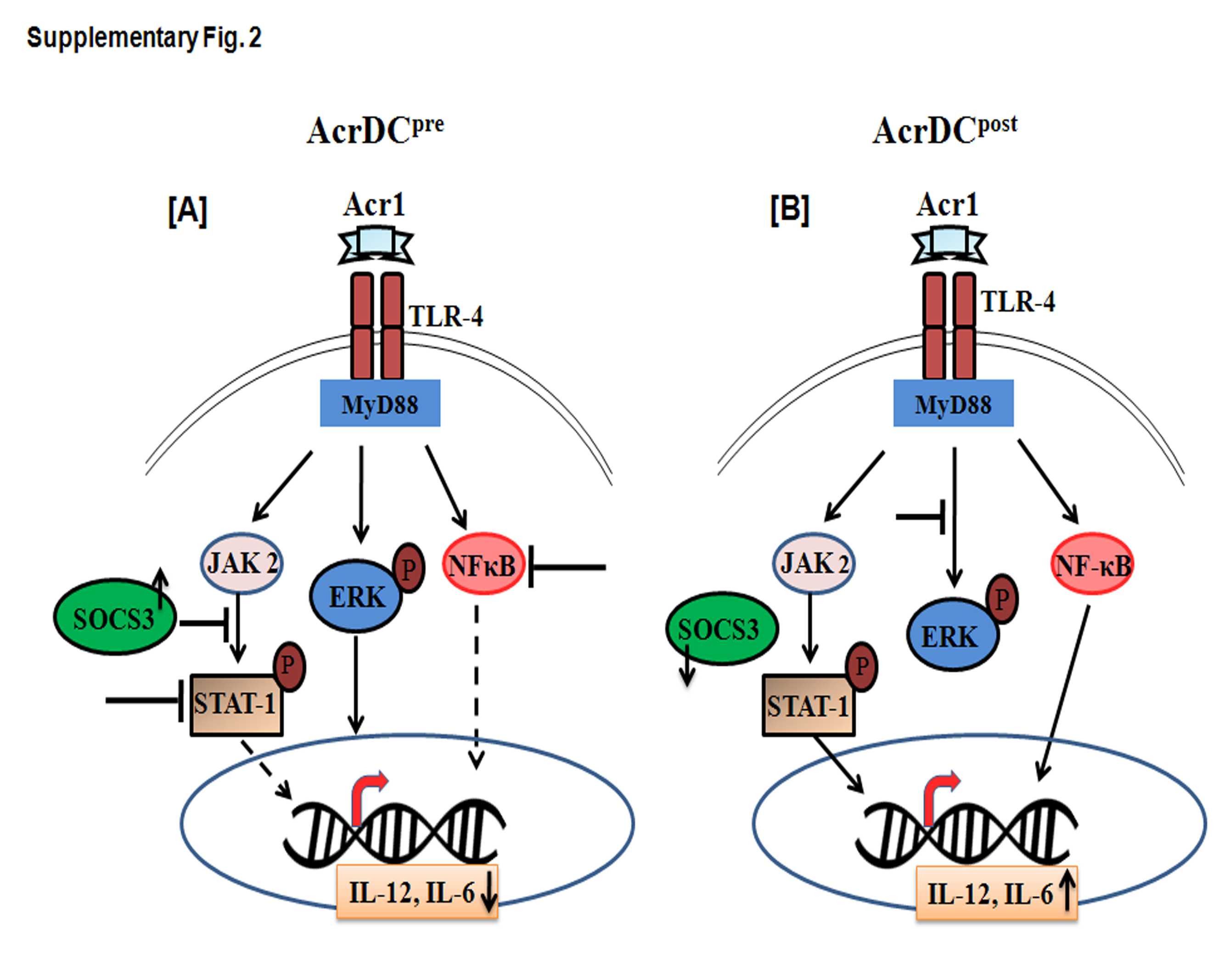

Supplement: Figure S2 — Alpha-crystallin protein (Acr1) differentially regulates the functionality of DCs. (A) During the differentiation, Acr1 abrogates the activation and maturation of DCs. In AcrDCpre, Acr1 suppresses the maturation of DCs by upregulating SOCS-3 which in turn inhibits activation of STAT-1. Moreover in AcrDCpre the phosphorylation of ERK is promoted while the translocation of NF-κB is hampered, leading to suppressed expression of proinflmmatory cytokines. (B) In contrast, Acr1 interaction with TLR-4 after maturation leads to SOCS-3 downregulation and enhanced activation of STAT-1. Furthermore, it also leads to downregulation of phosphorylation of ERK and facilitates the nuclear translocation of NF-κB. The activation of NF-κB leads to the upregulation of proinflammatory cytokines. [file Image_2.tif]
